# Hospitalization of Children with Down Syndrome

**DOI:** 10.3389/fpubh.2014.00022

**Published:** 2014-03-20

**Authors:** Ariel Tenenbaum, Rana N. Hanna, Diana Averbuch, Isaiah D. Wexler, Maor Chavkin, Joav Merrick

**Affiliations:** ^1^Pediatric Division, Hadassah Hebrew University Medical Center, Jerusalem, Israel; ^2^Department of Ophthalmology, Hillel Yaffe Medical Center, Hadera, Israel; ^3^Division for Intellectual and Developmental Disabilities, National Institute of Child Health and Human Development, Health Services, Ministry of Social Affairs and Social Services, Jerusalem, Israel; ^4^Kentucky Children’s Hospital, University of Kentucky, Lexington, KY, USA

**Keywords:** Down syndrome, children, child development, hospitalization, Israel

## Abstract

**Introduction:** Children with Down syndrome present with multiple medical problems in a higher prevalence compared with the general population, which may lead to hospitalizations.

**Methods:** Analysis of 560 hospitalizations of 162 children aged 0–16 years with Down syndrome at Hadassah Medical Center during the years 1988–2007 compared with data on children in the general population, hospitalized at the same period. Data was collected from patient files and statistical data from the Ministry of Health.

**Results:** Respiratory infections were the leading cause for hospitalization of children with Down syndrome. The number of hospitalizations of children with Down syndrome compared to the number of all children, who were hospitalized was surprisingly similar to their proportion in the general population. Eleven children died during their hospitalization (five heart failure, three sepsis, one respiratory tract infection, and one due to complication after surgery). Nine of the 11 had a congenital heart anomaly.

**Conclusion:** Children with Down syndrome can present with complex medical issues and we support the concept of a multidisciplinary team that has experience and knowledge to serve as a “one stop shop” for these individuals and their families, with timely visits in which a comprehensive evaluation is performed, problems attended to and prevention plans applied. In this way, we may prevent morbidity, hospitalizations, and mortality.

## Introduction

Down syndrome is the most prevalent genetic disorder in humans and the most common genetic cause for intellectual disability. Ninety-six percent of cases are the result of trisomy 21. According to the Ministry of Health records 6,900 individuals with DS live in Israel. The annual number of newborns in Israel is today 162,000, of which 160 have DS. This syndrome may cause multiple congenital anomalies, e.g., heart malformations, intestinal atresia, cataract, hearing loss, and hypothyroidism, as well as evolving medical conditions, e.g., diabetes mellitus, convulsions, and dementia (1–3). These medical issues need to be observed and treated adequately and continuously in order to prevent deterioration, loss of functions, hospitalizations, and fatalities.

Hilton et al. (4) in a study on respiratory disorders in children with DS found respiratory infections the leading cause for hospitalizations among 232 children with DS, mainly pneumonia and bronchiolitis, with hospitalizations two to three times longer than among children without DS. A survey (5) among parents to children with DS found that 88% of their children were hospitalized at least once, while Bloemers et al. (6) found a correlation between DS and severe respiratory syncytial viral (RSV) bronchiolitis.

Another study by So et al. (7) followed 200 newborns with DS and found that 54% were already hospitalized by the age of 3 years, mainly due to respiratory infections with congenital heart anomaly as a major risk factor for hospitalization. In an Israeli study, Megged and Schlesinger (8) reviewed medical records of all children with DS hospitalized in their center due to infection with respiratory syncytial virus during a 9 year period and found 41 hospitalizations of 39 children with DS with a mean age of 1.3 years and an average length of hospitalization of 10.9 days. Children with DS were older than healthy controls with respiratory syncytial virus infection and needed longer hospitalization.

The present study is concerned with the hospitalizations of children with DS hospitalized at two major medical centers in Jerusalem during 1997–2008. The morbidity, mortality, duration of hospitalization, population characteristics are described as well as compared with the general pediatric population hospitalized at these centers during the same years.

## Materials and Methods

This is an analytic retrospective study, based on 560 medical records of 162 children (age 0–16 years) with Down syndrome hospitalized in the Hadassah Medical Center during 1997–2008. Hadassah Hebrew University Medical Center includes two University Hospitals, Hadassah Ein Kerem and Hadassah Mount Scopus with a total of 1,000 beds. It is a referral center and a level 1 trauma center.

Using the computerized system of the hospital’s archives, we reviewed data from all discharge notes that included the diagnosis “Down syndrome” or “Trisomy 21,” regarding gender, ethnicity, age at hospitalization, length of stay, causes of hospitalization, co-morbidities, complications, or death. The number of hospitalizations of children with DS was compared with the number of hospitalizations of all children at the same period. The study was approved by the Institutional Review Board.

### Statistics

In order to compare quantitative variables one sample *t*-test was used. Comparing categorical data (e.g., prevalence of co-morbidities) one sample chi-square test was used. Testing the relation between two categorical data was done using chi-square test of Fisher’s exact test. Comparing quantitative variables (e.g., average length of stay per male vs. female) was done using *t*-test for two independent groups and Mann–Whitney test for abnormal distribution. All tests were bidirectional and a *P* value of 0.05 or less was considered statistically significant.

## Results

During the years 1997–2008 there were 255,764 hospitalizations of children in the Hadassah Medical Center. Of these 560 (0.22%, 1/457) were children with DS. Our population included a total of 162 children with DS who had records of 560 hospitalization episodes. One hundred sixteen (71.6%) were Jewish and 46 (28.4%) Arab. Twenty-nine percent had one record of hospitalization, 22% had two, and 49% had three or more hospitalizations. There was a male predominance of 62%. The main causes for hospitalization are detailed in Table 1, the causes of hospitalization by age in Table 2, and the causes of recurrent hospitalizations shown in Table 3.

**Table 1:** **Main causes for hospitalization**.

Cause	Number (%)	Mean age in months	Mean duration of hospitalization in days
Respiratory tract infections	222 (39.6)	36.76	7.62
Surgical procedures	125 (22.3)	30.29	9.65
Heart failure	45 (8)	27.52	12.62
Acute gastroenteritis	39 (7)	16.39	6.15
Sepsis	28 (5)	29.02	20.21
Failure to thrive	22 (3.9)	7.56	7.41
Jaundice in neonate	4 (0.7)	0.65	6.25
Urinary tract infections	3 (0.5)	39.33	3
Others	72 (12.9)	55.22	8.14
Total	560	33.76	9.03

**Table 2:** **Causes of hospitalization by age**.

Cause of hospitalization	*N*	Mean age (months)
Hyperbilirubinemia	4	0.65
Sepsis	27	29.02
FTT	22	7.57
Respiratory infection	222	36.76
Operation	125	30.29
Cardiac failure	45	27.52
Diarrhea	39	16.39
UTI	3	39.33
Other	72	55.23
Total	559	33.76

**Table 3:** **Causes of recurrent hospitalizations**.

Main frequent reason for hospitalization	Total (%)
Sepsis	4.32
FTT	4.32
Respiratory infection	55.56
Operation	19.75
Cardiac failure	3.7
Diarrhea	3.09
Other	9.26
Total	100

Respiratory tract infection was the main cause for the first recorded hospitalization in 55.6% of cases, and 55.56% in the second or third hospitalization.

Regarding the medical background of these children, including a history of heart anomalies, pulmonary hypertension, leukemia, gastrointestinal malformation, hearing impairment, adenoid hypertrophy, and hypothyroidism, we found no statistical significant difference between having any of those medical problems and the main reason of hospitalization. Of all of the above the leading reason was respiratory tract infections. There was no correlation between gender and/or ethnicity and a different main cause for hospitalization – again, respiratory infections were the leading cause in all sectors and genders.

Eleven children with DS died during their hospitalization, five had a severe heart anomaly and suffered from severe heart failure. Three died from severe sepsis, one from respiratory tract infection and failure, and one due to complication after surgery. Nine of the 11 had a congenital heart anomaly.

## Discussion

This study presents the main causes of hospitalizations and re-hospitalizations of children with Down syndrome aged 0–16 years. Our study showed that the leading cause for hospitalization was respiratory infections. A limitation of this study is the fact that it may not necessarily represent the general DS population in Israel. However, the male predominance and the prevalence of heart anomalies in our cohort, which is very similar to that reported on the DS population suggests that it may not be very different from the whole DS population in Israel.

We demonstrated that the duration of hospitalization for a child with DS who had a respiratory infection was on average 9 days, whereas sepsis may take as long as 20 days of hospitalization.

As mentioned above during 1997–2008, there were 255,764 hospitalizations of children in the Hadassah Medical Center with 560 (0.22%, 1/457) children with DS. According to information from the Ministry of Health the birth rate of neonates with DS is approximately 0.1% or 1 per 1,000. The total population of Israel is 7.8 million with 900,000 (11.5%) living in the Jerusalem region. In the years 1995–2003, a total of 1,155 children with DS were born in Israel, of those 323 (27.96%) in the Jerusalem district, which is more than double of the expected with regards to the population size of the Jerusalem district. This is probably due to the large proportion of ultra orthodox Jewish and Arab population in the city, who are not doing prenatal diagnosis. Therefore the proportion of children with DS, who were hospitalized was double to its proportion in the general population. In this population, good community services and well monitoring of their medical condition in an event of acute illness by their physicians is an important aspect. Nevertheless 49% of the children in our group were hospitalized three or more times. This again stresses the need for regular follow-up and surveillance of these children.

We found a strong relationship between DS and hospitalizations due to respiratory infections and therefore support the need for RSV, pneumoccocal, and influenza vaccines in this population.

In our study group, there were 11 fatalities, of which nine had a congenital heart anomaly. Although it is a small group, we may reasonably estimate that heart anomalies are risk factors for death in this population.

## Conclusion

In summary, morbidity of children with Down syndrome causes hospitalizations, which may be recurrent, frequent, and prolonged. It should be our goal as medical health providers to prevent or at least minimize as much as possible the medical problems among this special population. We support the concept of a multidisciplinary team that has experience and knowledge, and serves as a “one stop shop” for these individuals and their families (9–11), with timely visits in which a comprehensive evaluation is done, problems are attended to and prevention plans applied. In this way, we may prevent morbidity, hospitalizations, and mortality. This study emphasizes the need for comprehensive vaccinations against respiratory tract pathogens in this risk population, including anti pneumococcal vaccine, anti influenza vaccine, and anti RSV immunoglobulins.

## Conflict of Interest Statement

The authors declare that the research was conducted in the absence of any commercial or financial relationships that could be construed as a potential conflict of interest.
